# Teaching histology and anatomy online during the COVID‐19 pandemic

**DOI:** 10.1002/ca.23806

**Published:** 2021-11-10

**Authors:** Daniele Saverino, Emanuela Marcenaro, Daniela Zarcone

**Affiliations:** ^1^ Department of Experimental Medicine, Section of Human anatomy Università of Genoa Genoa Italy; ^2^ Laboratory of Autoimmunology Ospedale Policlinico San Martino Genoa Italy; ^3^ Department of Experimental Medicine, Section of Histology Università of Genoa Genoa Italy

**Keywords:** education, histology, human anatomy, sport science students

## Abstract

The aim of this study is to analyze differences in participation, and in the results obtained in the anatomy and histology exams, over two academic years of the Sport Sciences degree course. During the first semester of the academic year 2019/2020 both the lectures and the exam took place face‐to‐face, while during the academic year 2020/2021 everything was done online. Statistical analysis revealed that the online modality was especially advantageous for the anatomy exam. Students' opinions were also assessed through a short questionnaire. The results showed that teachers involved themselves in both groups. Students needed to interact socially with teachers and colleagues and to ask them questions. Even if the differences were not significant, the difference was greater for face‐to‐face students in most comparisons. Finally, the most common methods of peer communication were by social media.

## 1 INTRODUCTION

This article compares the scores in histology and human anatomy exams of students who received face‐to‐face lessons with those who received online lessons. The aim is to assess whether online training activities in histology and anatomy during the pandemic have been effective and whether a virtual learning community has been successfully created.

The Sport Science degree course in the faculty of medicine comprises 3 academic years plus a further 2 years for the master's degree. Histology and human anatomy are basic subjects taught in the first year of the course. Human anatomy teaching is divided into two semesters. In the first, anatomy excluding the nervous system is taught along with histology. In the second, the nervous system is presented. At the end of the first semester, which runs from October to December, students can start taking part of the exam during January and February. They complete the exams in the summer months with neuroanatomy.

On January 30, the World Health Organization declared COVID‐19 a global health emergency (Sohrabi et al., 2020). In March 2020, the Italian Government decided on a national blockade because of the COVID‐19 pandemic, which stopped face‐to‐face teaching in schools of all grades, including universities (DPCM 8‐3‐2020 OJ No. 59 8‐3‐2020; DPCM, 2020). In the University of Genoa, like others around the world, face‐to‐face lessons ceased. Efforts were therefore made to develop remote and/or virtual alternatives to continue teaching (Allsop et al., 2020; Attardi et al., 2018; Evans et al., 2020; Grainger et al., 2020; Stewart et al., 2011; Trelease, 2015; Triepels et al., 2020). The University of Genoa immediately organized itself to support teachers in organizing online courses.

Thus, while students attended face‐to‐face lessons and exams during the 2019/2020 academic year, all lessons were online using the Teams platform during the 2020/2021 academic year owing to the COVID‐19 pandemic. The exams in January and February were also held online, and this made it possible to schedule additional exam sessions during the winter session.

During this period, there was great concern among histology and anatomy teachers, particularly concerning the loss of face‐to‐face practical lessons. Histology and anatomy teachers faced several challenges including time, resources, technical skills, and educational innovation, which have helped to support learning by our students during the COVID‐19 emergency period.

## 2 MATERIALS AND METHODS

### 2.1 Course organization

**FIGURE 1:**
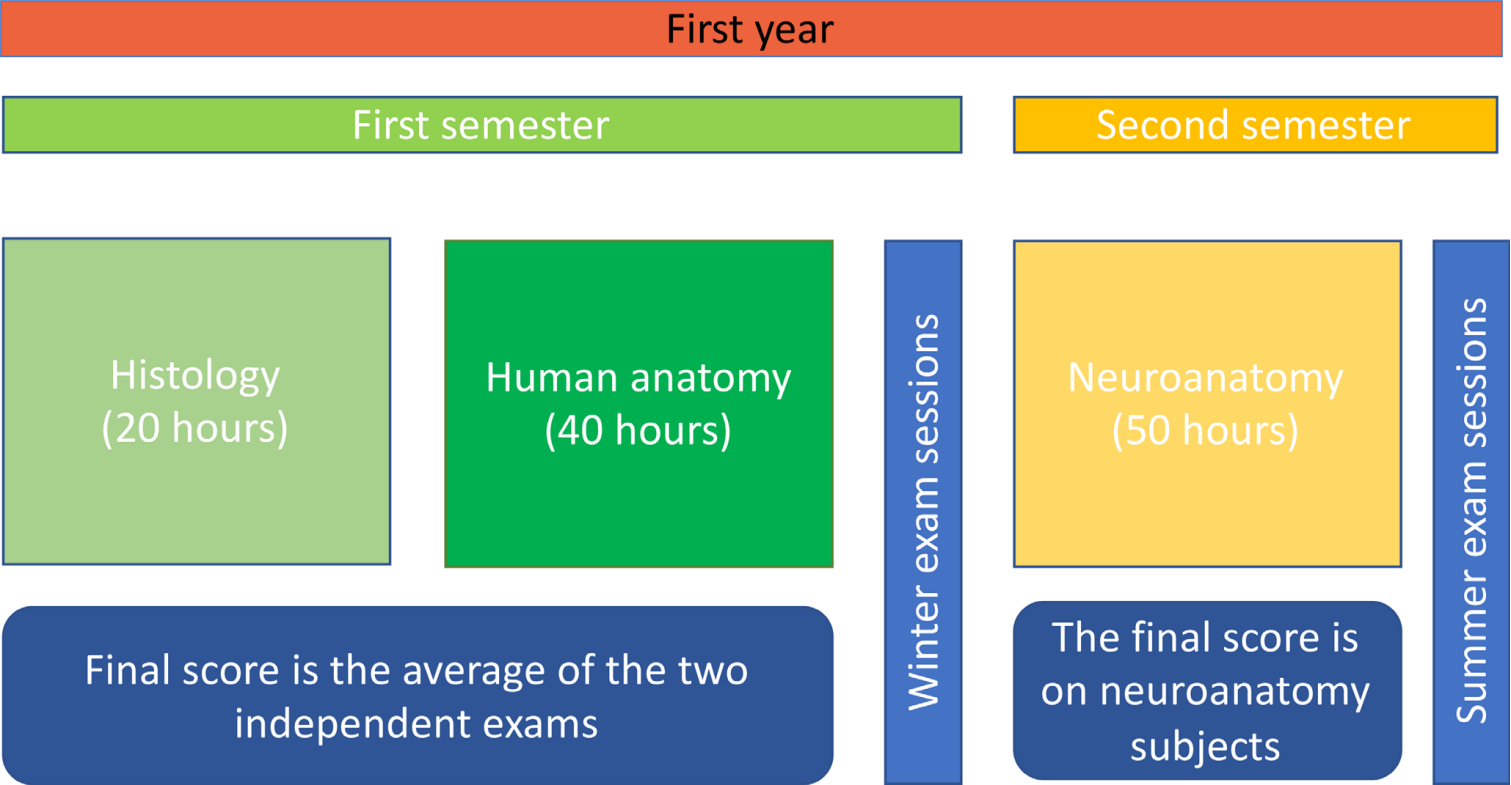
Outline of the organization of the course of anatomy and histology. Lessons in Histology and Human Anatomy (Locomotor Apparatus and Splancnology) are held during the first semester of the first year. During the second semester of the first year, there are lessons in Neuroanatomy. Students can take the first part of the exam in the winter session (January, February), while the Neuroanatomy part is examined in the summer session (June, July). The final grade is based on the weighted average for the entire course of studies

The number of students enrolled in the first of the two academic years was 147 in 2019 and 181 in 2020. Online teaching was designed to remain in line with the traditional face‐to‐face curriculum. For both histology and anatomy, the teacher explains topics using images, drawings, and videos; the same teaching method was used both face‐to‐face and online. There is no practical or microscopic part in this degree program. The examination is also carried out orally through a description of the subject, and there is no practical part. The same numbers of hours were provided in face‐to‐face and online lessons: 20 h for the histology course, which started earlier, and 40 h for the anatomy course (musculoskeletal system and splanchnology), which started a few weeks later and ended in December. In January and February, students accessed the first exam sessions (covering histology and the first part of anatomy) (Figure 1). Marks/scores were established by the Academic Rules and Regulation for Italian Universities. The mark is expressed out of 30 (the minimum pass score is 18/30, while the maximum is 30/30 cum laude). One mark is given for histology and one for anatomy, and the weighted average is obtained from the number of credits in the two disciplines.

### 2.2 Students' perceptions

The opinions of the students of both academic years were obtained via a short questionnaire. Items from the previous study (Attardi et al., 2016) were adapted for the present one (see Supplement material). Responses were given on a five‐point Likert scale (5 = strongly agree, 4 = agree, 3 = uncertain, 2 = disagree, and 1 = strongly disagree) (Point Likert scale, 2010).

### 2.3 Statistical analyses

The normality of the distribution of data was verified by the D'Agostino‐Pearson normality test (Stephens, 1986). This test computes skewness and kurtosis (K‐S test) to quantify how far the distribution is from Gaussian in terms of asymmetry and shape; it then calculates how far each value differs from that expected with a Gaussian distribution, and computes a single P value from the sum of these discrepancies. For the 2019/2020 cohort the K‐S test results for histology and anatomy were 0.1393, *p* = 0.1514, and 0.1695, *p* = 0.7918, respectively. For the 2020/2021 cohort the K‐S test results for histology and anatomy were 0.1359, *p* = 0.0642, and 0.1818, *p* = 0.1097, respectively.

The paired two‐sample, two‐tailed *t* test was used for statistical analysis. A *p* value less than 0.05 was considered statistically significant. The median, upper, and lower limits of the 95% confidence interval for both the difference and the mean were also recorded. The final score 30 cum laude was counted as 32 and then used for the statistical analysis. GraphPad Prism software 6.0 (GraphPad Software Inc., CA) was used for all the analyses.

Nonparametric, one‐way ANOVA (Kruskal–Wallis test) followed by Dunn multiple comparisons test was used to compare the students' opinion data.

## 3 RESULTS

Final examination scores were collected during the winter session from students over two academic years. Those in the 2019/2020 cohort followed the histology and human anatomy course face‐to‐face, those in the 2020/2021 cohort by telematic lessons and exams.

**FIGURE 2:**
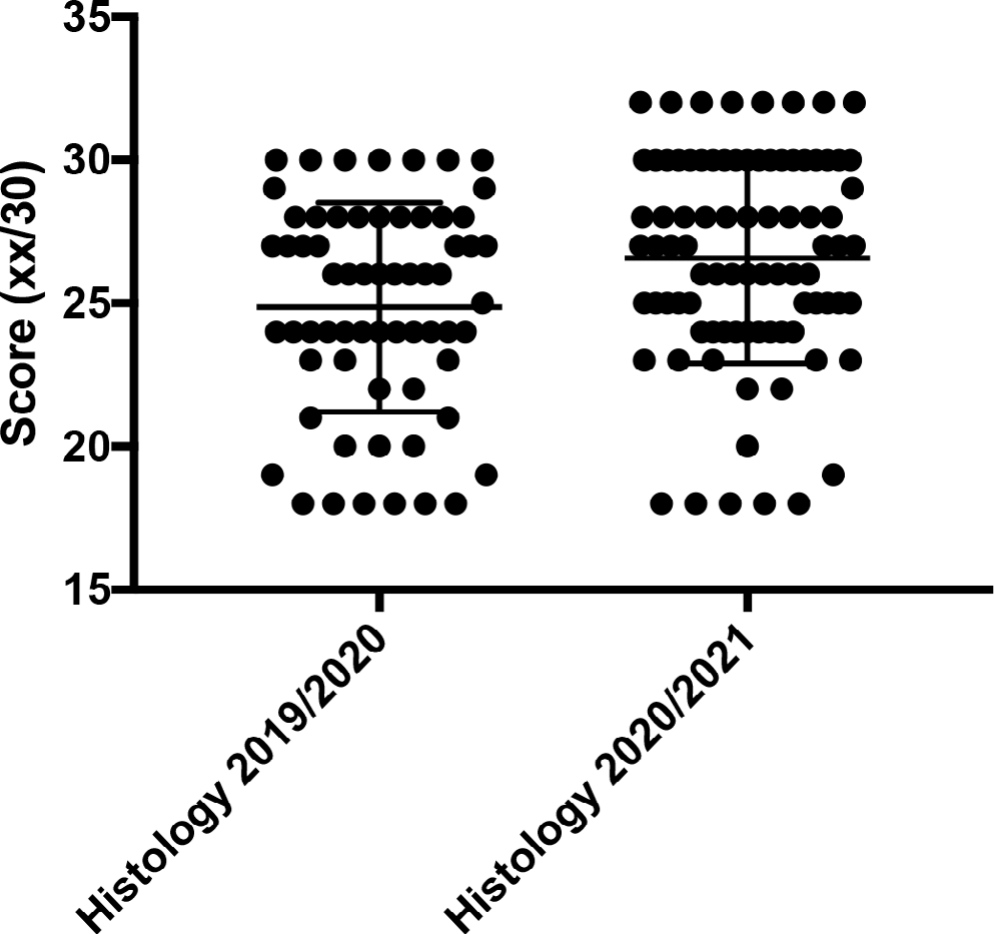
The online group obtained a better final histology score (*p* = 0.0063)

**FIGURE 3:**
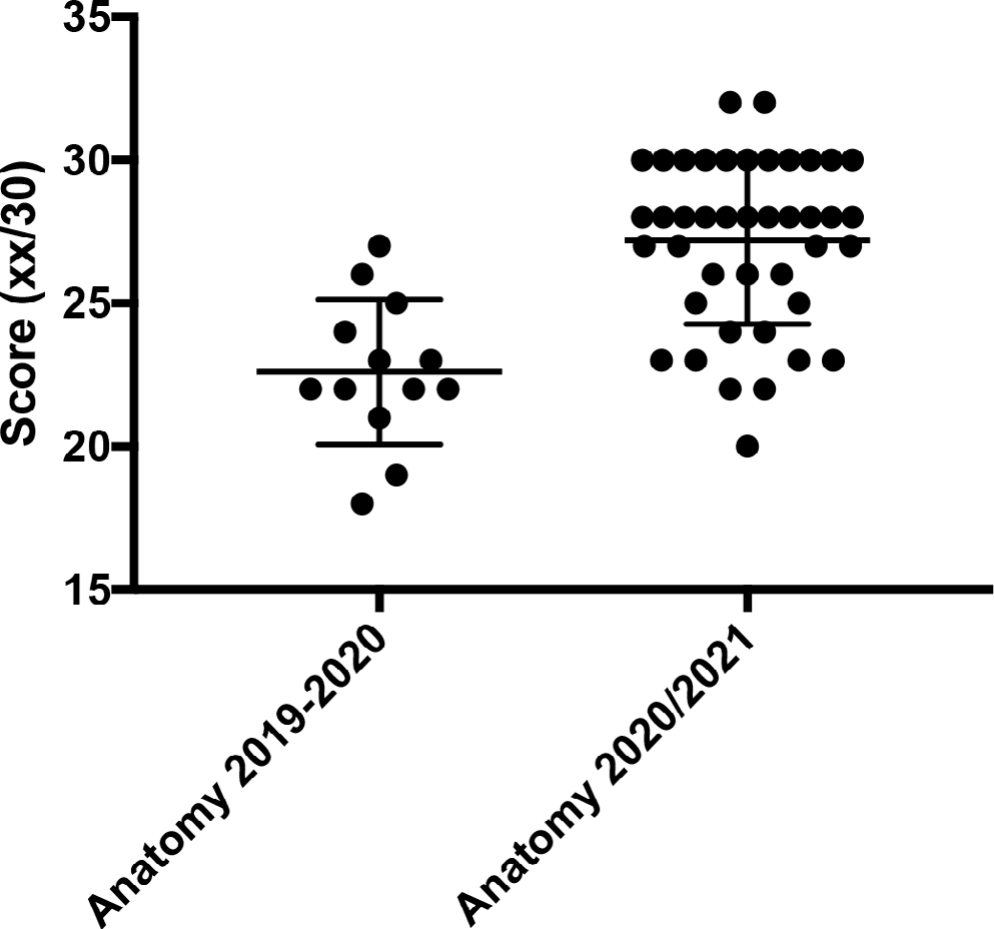
The online group obtained a better final anatomy score (*p* < 0.0001)

Among the students who took face‐to‐face lessons, 65 out of the total of 147 (44.2%) passed the histology exam; 37 did not score positively in the examination (25.2%). In addition, 13 students (8.8%) scored the anatomy exam positively during the winter session (16 in total were evaluated unsuccessfully, 10.9%). In contrast, among students who attended the online lessons, 88 out of the total of 181 (48.6%) scored positively the histology—31 did not score positively in the examination (17.1%)—and 42 (23.2%) in anatomy. In addition, 29 out of 181 did not score positively in the anatomy examination (16.0%). Thus, it can be deduced not only that students who attended online lessons obtained better grades, but also that fewer of those students had negative final grades than those who took the F‐2‐F lessons. The data are summarized in Figures 2 and 3. In both the academic years 2019/2020 and 2020/2021, only the students who took and passed the exam were considered.

The final examination scores were then analyzed (total/30). The interesting finding is that the online group obtained better scores in both histology (*p* = 0.0063) and anatomy (*p* < 0.0001). The results are summarized in Table 1.

**TABLE 1:** Final scores (xx/30) of anatomy examinations per academic year

	Histology 2019/2020	Histology 2020/2021	Anatomy 2019–2020	Anatomy 2020/2021	Histology + anatomy 2019/2020	Histology + anatomy 2020–2021
Number of values	64	86	13	42	13	41
Minimum	18	18	18	20	18.5	21
25% percentile	23	24	21.5	25	23.5	25
Median	26	27	22	28	24.5	27.5
75% percentile	28	30	24.5	30	26.25	30
Maximum	30	32	27	32	27.5	32
Mean	24.86	26.58	22.62	27.19	24.23	27.27
*SD*	3.647	3.673	2.534	2.924	2.766	2.875

As can be seen from Figures 1 and 2, the results were statistically significant for histology but much more significant for anatomy, in both the number of students who presented themselves for the exam and the number who passed it.

**FIGURE 4:**
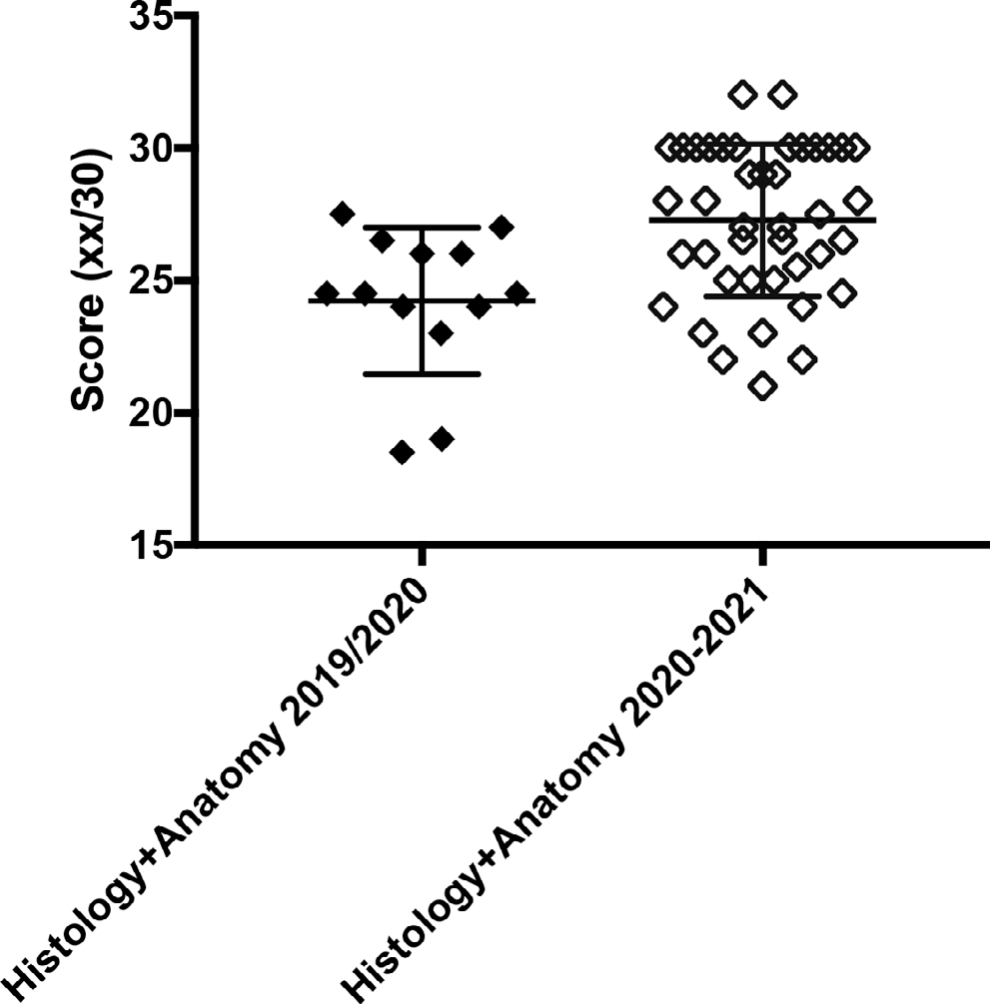
Analysis of the final histology and anatomy scores (average of the two independent scores) shows that the online group obtained a better final score (*p* = 0.0024)

Finally, considering the average results for the histology and anatomy exams, it is statistically evident that the students who followed the online lessons obtained better results (*p* = 0.0024) (Figure 4).

From the short questionnaire, it emerged that the students were satisfied with the online lessons because they could interact with the teacher at any time, but above all because recorded lessons were available for them to listen to repeatedly, improving their understanding and the final results. On average, most students in both years agreed, using the five‐point Likert scale, that they were able to interact socially with professors (4.4 ± 0.38 face‐to‐face and 4.19 ± 0.56 online) and ask questions (4.20 ± 0.37 face‐to‐face and 3.97 ± 0.71 online). These results were not statistically different. In addition, the students agreed that they were involved by the professors during the lesson (4.30 ± 0.51 face‐to‐face and 3.94 ± 0.76 online). They obviously felt more involved during face‐to‐face lessons; however, these differences were not statistically significant, thanks to the teachers' commitment during the pandemic period.

**FIGURE 5:**
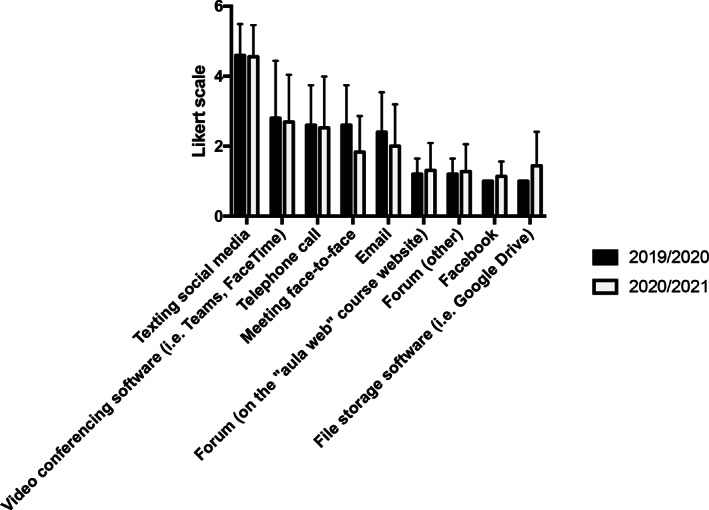
Frequency of student–student communication methods reported as mean ± *SD*

Student–student contact proved to be mainly via messaging on social media (4.60 ± 0.90 face‐to‐face, and 4.56 ± 0.91 online); for results see Figure 5. Again, no significant differences between the two groups were evident except in regard to meeting face‐to‐face (2.83 ± 1.47 face‐to‐face and 1.67 ± 0.85 online; *p* = 0.029). Obviously, the group of students who followed the lessons online during the pandemic had limited ability to meet each other (DPCM 8‐3‐2020 OJ No. 59 8‐3‐2020).

## 4 DISCUSSION

The COVID‐19 pandemic forced universities to move their courses online to avoid interrupting the continuity of study preparation (Allsop et al., 2020; Attardi et al., 2018; Evans et al., 2020; Franchi, 2020; Grainger et al., 2020; Green et al., 2014; Iwanaga et al., 2021; Trelease, 2015). At the end of this experience in the first semester of the Sport Science course, we asked what learning results we had obtained and how the students perceived the effectiveness of this modality of teaching, comparing two academic years during which students had followed face‐to‐face or online lessons.

Analysis of the data showed that the online mode proved advantageous for students enrolled in the first year of the Sport Science degree course. The reasons are various:

1. Students could have used the traveling time between home and university to study.
2. Recorded lessons could be delivered both synchronously and asynchronously; in any case, it was possible to listen to the recording of each lesson several times, clarifying the topic and deepening understanding.
3. It was easier to arrange meetings with teachers online to seek explanations.
4. The teacher was more willing to schedule more exam sessions.

The disadvantages for students were mainly linked to the lack of interaction among them, and the disadvantage for teachers was the impossibility of real and immediate perception of student involvement and attention (Johnson et al., 2013; Murphy et al., 2008). The Sport Science course also contains a conspicuous practical part that cannot be carried out online. This was disadvantageous during the lockdown and probably left more space for the study of basic subjects such as histology and anatomy.

Students noted a big difference between theoretical courses and courses that require practical exercises. In the latter, of course, the need for face‐to‐face lessons is much greater.

The appreciation of online lessons is an important result to have obtained during this pandemic period. In addition, it seems that smartphones and electronic devices have served as tools for the dissemination of information, activity, and communication during times of physical distancing. Digital infrastructures are not free from problems; primarily, they can contribute to the spread of disinformation (Singh et al., 2020). However, careful and informed use of mobile social platforms (such as Facebook, Twitter, and WhatsApp) shows that physical distance does not necessarily lead to disinformation. It would therefore appear that in the event of a pandemic, digital media can be effective for helping individual students overcome difficulties. However, interaction through digital media cannot be held responsible for student outcomes during the pandemic time. These media are probably surrogates for face‐to‐face interaction.

The experience of this period could be important for evaluating the possibility of inserting online lessons into a mostly face‐to‐face teaching course to facilitate discussion and clarification of complex issues. Another possibility could be to provide recordings of material in advance in order to use innovative methods during face‐to‐face sessions, such as videos of the anatomy of movement and 3D anatomical models. Thus, we are confident that these challenges will provide opportunities to improve the quality of our current face‐to‐face teaching when the situation finally returns to normal (Franchi, 2020; Iwanaga et al., 2021; Nicholson et al., 2006).

## 5 CONCLUSIONS

Although the restrictions and limitations due to the pandemic have been difficult for both teachers and students to deal with, it has been possible to conduct the study program online as if it were in the classroom. The advantages of online teaching have been described in the discussion and this has allowed students to obtain better results in the exams, at least as regards anatomy and histology.

Our intention is not to emphasize online teaching on the strength of these findings, but to promote the use of resources that can help students in studying such a broad and complex subject, or support students who are unable to follow the course because they are engaged in off‐site problems, for example. The use of tools such as recorded lessons or online meetings with the teacher could continue even when the pandemic is resolved and traditional lessons return.

## Supporting information


**Appendix** S1: Supporting Information
