# Supplementary material for: Teaching histology and anatomy online during the COVID‐19 pandemic
Source: Clin Anat. 2021 Nov 10;35(1):129–34. doi: 10.1002/ca.23806 (PMC8653158; doi:10.1002/ca.23806)
Supplement: Supplementary file 1 — Appendix S1: Supporting Information [file CA-35-129-s001.docx]

**Supplement 1.**

Survey Questions

| **PART 1: DEMOGRAPHIC INFORMATION** | |
| --- | --- |
| 1. What is your age in years? ___ | |
| 1. What is your gender? 2. Male 3. Female 4. Other 5. I prefer not to answer | |
| 1. Academic year: 2. 2019/2020 3. 2020/2021 | |
| **PART 2: EVALUATION OF LEARNING EXPERIENCE**  **Questions for Online Students Questions for Face-to-Face Students** | |
| 4. Which of the following statements best describes how you attended your lessons?   1. I followed most of the lessons by accessing during the synchronous lessons 2. I followed most of the lessons by accessing the asynchronous recorded lessons 3. I followed some of the lectures in synchronous, and some in asynchronous 4. Other. Please describe. ____________ 5. I prefer not to answer | *Question not applicable to face-to-face students* |
| 5. Below are the statements related to your online lessons. Please evaluate how much you agree with each statement. *Likert scale: strongly disagree, disagree, neither agree nor disagree, agree, strongly agree*   1. I am able to interact socially with my peers online 2. I am able to ask my online peers questions about the course 3. I am able to interact socially with my professors 4. I am able to ask the professor my questions 5. I feel involved with professors when I attend my online lessons 6. It is important to me to be able to see professor on video during lesson 7. I appreciate the ability to watch the recordings of the lessons whenever I want 8. I appreciate the ability to fast forward, rewind and pause the recordings of the lessons 9. I find that I have often delayed viewing the lessons because I know they are available to me at any time | 5. Below are the statements related to your online lessons. Please evaluate how much you agree with each statement. *Likert scale: strongly disagree, disagree, neither agree nor disagree, agree, strongly agree*   1. I am able to interact socially with my peers online if I choose it 2. I am able to ask my peers questions about the course 3. I’m able to interact socially with my professors 4. I am able to ask the professor my questions 5. I feel involved with professors when I attend my face-to-face lessons |
| **PART 3: PEER COLLABORATION**  **Questions for Online Students Questions for Face-to-Face Students** | |
| 6. Please evaluate how often you communicate with your classmates regarding the course (i.e. to ask a question, to study) using the following means of communication. *Likert Scale: never, rarely, occasionally, a moderate amount, a great deal*  Email  Forum (on the OWL course website)  Forum (other)  Meeting face-to-face  Telephone call  Video conferencing software (e.g. Skype, FaceTime)  Texting Social media (e.g. SMS text, iPhone Instant Messenger, Black Berry Messenger)  Facebook  File storage software (e.g. Google Drive) | |
